# Development and validation of a frailty risk prediction model in patients with peripheral artery disease

**DOI:** 10.3389/fsurg.2025.1682178

**Published:** 2026-01-16

**Authors:** Qingmei Fang, Fengwang Xue, Xueshuang Chen, Xia Qing, Feng Liu, Shengmin Guo

**Affiliations:** 1 Department of Vascular Surgery, The Affiliated Hospital of Southwest Medical University, Luzhou, China; 2 School of Nursing, Southwest Medical University, Luzhou, China; 3 Department of Nursing, The Affiliated Hospital of Southwest Medical University, Luzhou, China

**Keywords:** frailty, nomogram, older adults, peripheral artery disease, predictive model

## Abstract

**Objective:**

To investigate the current status and risk factors of frailty among patients with peripheral artery disease, and to develop a risk prediction model to inform targeted clinical interventions.

**Methods:**

Patients were consecutively recruited for this investigation from August 2024 to May 2025. The study included 499 individuals with peripheral artery disease who were hospitalized in the vascular surgery department of a tertiary hospital in Southwest China. Data were collected using a general information questionnaire, laboratory test results, the Barthel Index, and the Social Support Rating Scale. The Tilburg Frailty Indicator was used to classify patients into a non-frailty group and a frailty group. The dataset was randomly split in a 7:3 ratio into a training set and a validation set. Independent predictors of frailty were identified through univariate and multivariate logistic regression analyses. The risk prediction model was developed using R software. Discrimination of the model was evaluated by plotting receiver operating characteristic (ROC) curves and calculating the area under the curve (AUC), sensitivity, and specificity in both the training and validation sets. Model calibration was assessed using the Hosmer–Lemeshow goodness-of-fit test and calibration curves. Clinical utility was evaluated using decision curve analysis.

**Results:**

Age, hemoglobin level, number of comorbidities, and activities of daily living were identified as independent risk factors for frailty. In the training set, the AUC was 0.771 (95% CI: 0.721–0.821), with a sensitivity of 0.788 and a specificity of 0.808. In the validation set, the AUC was 0.704 (95% CI: 0.620–0.788), with a sensitivity of 0.743 and a specificity of 0.682. The Hosmer–Lemeshow test indicated good calibration in both the training set (*χ*^2^ = 7.967, *P* = 0.435) and the validation set (*χ*^2^ = 9.642, *P* = 0.291). DCA showed that the model provided net clinical benefit within threshold probability ranges of 10%–80% in the training set and 20%–74% in the validation set.

**Conclusion:**

The developed risk prediction model exhibited predictive performance and can assist clinical healthcare providers in identifying populations at high risk of frailty among patients with PAD, thereby providing a reference for developing intervention strategies targeting relevant risk factors.

## 1 Introduction

Peripheral arterial disease (PAD) is a chronic, progressive condition characterized by the narrowing and occlusion of lower extremity arteries due to atherosclerosis (1). Patients with PAD may present with mild or no symptoms, intermittent claudication, or chronic limb-threatening ischemia (2). Epidemiological evidence indicates that PAD is more common in individuals aged ≥50 years, with prevalence increasing progressively with age; it rises markedly to approximately 9% among those aged 60–69 years and reaches 20% in individuals aged ≥80 years. Currently, an estimated 230 million people worldwide are living with PAD (3, 4). PAD is the third leading manifestation of atherosclerosis after coronary artery disease and cerebrovascular disease (5). These trends underscore the importance of strengthening the health management of patients with PAD (4).

Frailty is a non-specific clinical condition characterized by concurrent declines in multiple physiological systems, resulting in heightened vulnerability and reduced resilience to external stressors (6). The 2020 International Consensus Guidelines on Frailty highlight its association with a significantly elevated risk of adverse outcomes, including unplanned hospitalization, cognitive impairment, depression, and even mortality. Moreover, frail individuals incur 22% higher healthcare costs than their non-frail counterparts, reflecting the serious impact of frailty on physical and mental health (7). Previous studies have reported frailty prevalence rates of 26.1% and 49% among patients with PAD, identifying it as an independent predictor of poor prognosis (8, 9). In vascular surgery patients, frailty has been significantly associated with impaired walking ability, postoperative complications, major amputations, and increased mortality in individuals with PAD, severely compromising patients' quality of life and clinical outcomes (10–12).

Previous frailty prediction models developed for atherosclerotic diseases have primarily focused on populations with coronary artery disease and stroke (13, 14). In contrast, prior studies on peripheral vascular disease have mainly investigated the prevalence of frailty and its associated factors, with few attempts to develop risk prediction models to identify individuals at high risk of frailty. In the present study, we incorporated additional predictors—such as laboratory indices, activities of daily living, and social support—thereby providing a more comprehensive pathophysiological perspective and enhancing the interpretability of the model. Early warning and precise interventions are crucial for preventing or delaying frailty progression. Therefore, this study aimed to identify risk factors for frailty in patients with PAD using univariate and multivariate logistic regression analyses and to develop a visual nomogram-based risk prediction model, providing a quantitative assessment tool for clinicians and nurses and supporting clinical decision-making for frailty identification and prevention in patients with PAD.

## 2 Materials and methods

### 2.1 Study population and design

Patients with PAD who were consecutively admitted to the Department of Vascular Surgery at a tertiary hospital in Southwest China between June 2024 and May 2025 were enrolled. The inclusion criteria were: being conscious and able to communicate effectively; and meeting the diagnostic criteria for PAD (1): Typical clinical manifestations of intermittent claudication in patients with PAD, diminished or absent distal arterial pulses in the affected limb, a resting ankle–brachial index ≤0.90 in the affected limb, and imaging evidence of lower-extremity arterial stenosis or occlusion on color Doppler ultrasonography, computed tomography angiography, magnetic resonance angiography, or digital subtraction angiography. The exclusion criteria were psychiatric disorders and advanced malignancy or severe complications. Based on previous literature, 24 candidate predictors were considered (15–17). The sample size was calculated using the events-per-variable (EPV) method; to ensure stable logistic regression estimates, an EPV of 10 was adopted. Given a reported frailty prevalence of 55.5% among patients with end-stage PAD, the minimum sample size required for model development was estimated as (24 × 10/0.555) ≈ 436 (18). Allowing for a 10% attrition rate, at least 479 participants were required. Ultimately, 499 patients with PAD were enrolled, meeting the statistical requirements. This study was approved by the Ethics Committee of the hospital (Approval No.:KY2025403). All participants provided written informed consent and voluntarily participated in the study.

### 2.2 Data collection

#### 2.2.1 Research instrument

Predictor variables for frailty risk in patients with PAD were determined through a literature review, group discussions, and expert consultation. Relevant data were collected before hospitalization treatment. Demographic characteristics included: age, sex, body mass index (BMI), smoking status, education level, occupation, per capita household income, marital status, place of residence, living status, and type of medical insurance. Disease-related clinical data included: disease stage (Fontaine classification), limb symptoms, and number of comorbidities (comorbiditie including diabetes mellitus, hypertension, coronary artery disease, stroke, chronic kidney disease).

#### 2.2.2 Laboratory indicators

Fasting venous blood samples were collected from patients upon hospital admission. Laboratory parameters were measured using an automated biochemical analyzer, and data were retrieved through the hospital information system (HIS). The collected indicators included: total protein (g/L), albumin (g/L), prealbumin (mg/L), hemoglobin (g/L), white blood cell count (×10⁹/L), neutrophil count (×10⁹/L), lymphocyte count (×10⁹/L), and high-sensitivity C-reactive protein (hs-CRP, mg/L).

#### 2.2.3 Tilburg frailty indicator

The Tilburg Frailty Indicator (TFI), developed by Gobbens et al. in the Netherlands, is a self- or interviewer-administered instrument designed for rapid screening of frailty syndrome in older adults (19). It comprises 15 items across three domains: physical frailty (8 items), psychological frailty (4 items), and social frailty (3 items). Items 1–8, 12, 13, and 15 are scored dichotomously (“yes”/ “no”), with scores of 1 and 0, respectively. The remaining items use a three-point response format (“yes”/ “sometimes”/ “no”), scored as 1, 1, and 0, respectively. The total score ranges from 0 to 15, and a score of ≥5 indicates frailty. In the present study, the Cronbach's *α* coefficient of the TFI was 0.788.

#### 2.2.4 Activity of daily living

Activities of Daily Living (ADL) refer to the basic functional abilities required for an individual to maintain basic survival and independent living in daily life, reflecting one's capacity for self-care and overall functional status. The Barthel Index, developed by Mahoney and Barthel in 1965, is a widely used instrument for assessing basic ADL in older adults (20). It evaluates a patient's ability in ten domains: eating, bathing, grooming, dressing, bowel control, bladder control, toileting, transferring (bed to chair), walking on flat ground, and climbing stairs. The total score ranges from 0 to 100. A score of 100 indicates full independence; 60–99 indicates mild dysfunction; 41–59 indicates moderate dysfunction; and ≤40 indicates severe dysfunction. Higher scores reflect better self-care ability. In this study, the Cronbach's alpha coefficient for the ADL scale was 0.908, indicating high reliability and validity.

#### 2.2.5 Social support

Social support refers to the objective support, subjective support, and the degree of utilization of support that individuals obtain from family, friends, and their social networks in real life, reflecting the level of social support available to them and actually utilized. The Social Support Rating Scale (SSRS), developed in 1994 by the Chinese scholar Professor Xiao Shuiyuan, is used to assess individuals' social support (21). It comprises three domains—objective support, subjective support, and utilization of social support—across 10 items. The total score ranges from 11 to 72, with higher scores indicating greater levels of social support. The classification criteria are as follows: ≤22 indicates low-level social support, 23–44 indicates moderate-level, and ≥45 indicates high-level social support. In this study, the Cronbach's alpha coefficient for the SSRS was 0.709.

### 2.3 Questionnaire survey method

Relevant data were collected from patients prior to hospital admission for treatment. Investigators explained the purpose, significance, and instructions of the survey using standardized guidance. After obtaining informed consent, participants were given a general questionnaire (covering demographic characteristics) and the SSRS questionnaire. Patients completed the questionnaires independently; for those unable to read, investigators provided assistance. Questionnaires were collected immediately on-site, and completeness was checked, with patients prompted to address any missing items. Disease-related information and laboratory indicators were extracted by two researchers from the electronic medical record system. The Tilburg Frailty Indicator and Barthel Index were assessed and completed by the principal investigator. A total of 520 questionnaires were distributed, and 499 valid responses were collected, yielding a valid response rate of 95.9%.

### 2.4 Statistical methods

Data were analyzed using SPSS version 27.0 and R Studio version 4.1.3. The enrolled patients were randomly divided into two groups in a 7:3 ratio for model development and validation. Missing data were addressed using multiple imputation: predictive mean matching was used for continuous variables and logistic regression for binary variables. Normally distributed continuous variables are presented as mean ± standard deviation, whereas non-normally distributed variables are presented as median (Q1, Q3). Independent samples t-tests were used for normally distributed continuous variables, and the nonparametric Wilcoxon rank-sum test was applied otherwise. Categorical variables were expressed as frequencies or percentages, and group comparisons were conducted using the chi-square test or Fisher's exact test, as appropriate. Variables with *P* < 0.05 in univariate analyses were entered into a multivariable logistic regression model to identify independent predictors of frailty. A nomogram was constructed using the *rms* package in R. Model discrimination was assessed using receiver operating characteristic (ROC) curves and the area under the ROC curve (AUC). Model calibration was evaluated using calibration plots, the Hosmer–Lemeshow goodness-of-fit test, and the Brier score (<0.25). Decision curve analysis was performed to assess clinical utility. All statistical tests were two-sided, with a *P*-value < 0.05 considered statistically significant.

## 3 Results

### 3.1 Participant characteristics

**Figure 1:**
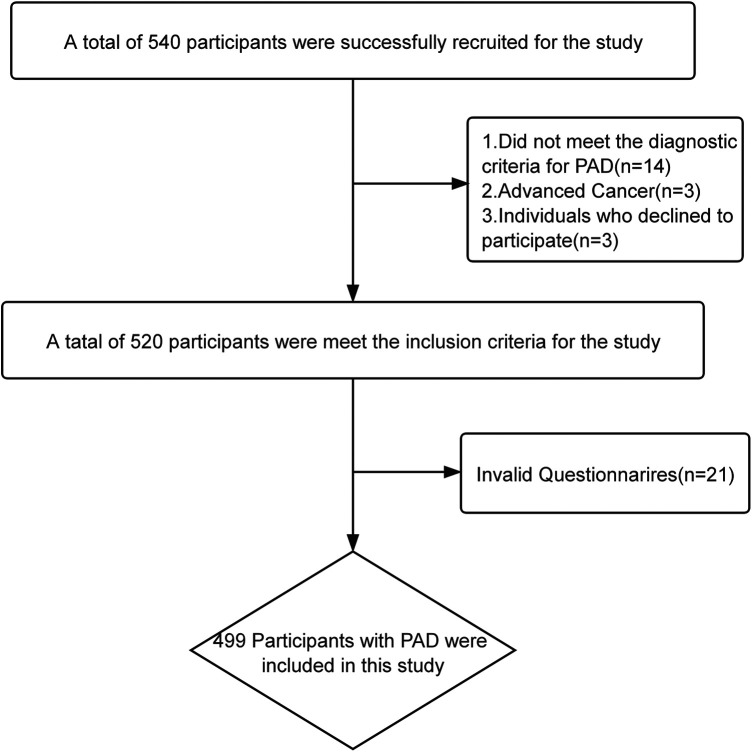
Flowchart of research participant recruitment.

A total of 499 patients with PAD were included in this study, and the participant recruitment flowchart is shown in Figure 1. Of these, 350 patients were assigned to the training set and 149 to the validation set. In the training set, there were 249 men and 101 women; the age ranged from 50 to 95 years, with a mean age of 74.37 ± 7.47 years. The prevalence of frailty in the training set was 43.4%, including 198 non-frail and 152 frail patients. The baseline characteristics of the training set and the results of the univariate analyses are presented in Table 1.

**Table 1:** Prevalence of frailty and related variables in patients with PAD (*n* = 350).

Variables	Non-frailty (*n* = 198^1^)	Frailty (*n* = 152^1^)	Statistic^2^	*P* value^2^
Sex			0.073	0.787
Male	142 (71.72%)	107 (70.39%)		
Female	56 (28.28%)	45 (29.61%)		
Age			11.793	0.003
50∼70	96 (48.48%)	48 (31.58%)		
71∼80	60 (30.30%)	52 (34.21%)		
>80	42 (21.21%)	52 (34.21%)		
BMI(kg/m^2^)			4.440	0.109
<18.5	15 (7.58%)	21 (13.82%)		
18.5∼24	124 (62.63%)	95 (62.50%)		
>24	59 (29.80%)	36 (23.68%)		
Smoking			0.013	0.910
No	95 (47.98%)	72 (47.37%)		
Yes	103 (52.02%)	80 (52.63%)		
Education level			2.143	0.342
Primary school and below	124 (62.63%)	106 (69.74%)		
Middle school	59 (29.80%)	35 (23.03%)		
College Degree or Higher	15 (7.58%)	11 (7.24%)		
Marital status			0.127	0.722
Married	74 (37.37%)	54 (35.53%)		
Umarried	124 (62.63%)	98 (64.47%)		
Occupational			2.160	0.340
Working	25 (12.63%)	19 (12.50%)		
Retirement	94 (47.47%)	61 (40.13%)		
Other	79 (39.90%)	72 (47.37%)		
Per Capita Household Income			4.237	0.120
<2,000	33 (16.67%)	33 (21.71%)		
2,000∼4,999	105 (53.03%)	87 (57.24%)		
≥5,000	60 (30.30%)	32 (21.05%)		
Residence area			2.719	0.257
Villages	69 (34.85%)	57 (37.50%)		
Town	84 (42.42%)	52 (34.21%)		
City	45 (22.73%)	43 (28.29%)		
Residential status			0.449	0.396
Living alone	21 (10.61%)	21 (13.82%)		
Communal living	176 (88.89%)	130 (85.53%)		
Medical insurance type			5.193	0.023
Urban employees	89 (44.95%)	87 (57.24%)		
Urban residents	109 (55.05%)	65 (42.76%)		
Pathological limb			2.862	0.239
Both lower limb	51 (25.76%)	51 (33.55%)		
Right lower limb	71 (35.86%)	45 (29.61%)		
Left lower limb	76 (38.38%)	56 (36.84%)		
Fontaine stage			9.488	0.024
Ⅱa stage	10 (3.03%)	5 (7.89%)		
Ⅱb stage	30 (12.63%)	15 (13.16%)		
Ⅲ stage	70 (40.40%)	40 (34.21%)		
Ⅳ stage	88 (43.94%)	92 (44.74%)		
Number of comorbidities			9.473	0.009
0	19 (9.60%)	3 (1.97%)		
1∼3	136 (68.69%)	106 (69.74%)		
>3	43 (21.72%)	43 (28.29%)		
ADL			8.976	0.030
Full independence	11 (5.56%)	1 (0.66%)		
Mild functional impairment	91 (45.96%)	60 (39.47%)		
Moderate functional impairment	53 (26.77%)	49 (32.24%)		
Severe functional impairment	43 (21.72%)	42 (27.63%)		
Social support			12.306	0.002
Low-level	95 (47.98%)	100 (65.79%)		
Moderate-level	30 (15.15%)	20 (13.16%)		
High-level	73 (36.87%)	32 (20.05%)		
TP (g/L)	66.25 (60.00, 73.00)	66.95 (60.15, 73.80)	−0.114	0.910
ALB (g/L)	40.85 (37.40, 45.00)	41.30 (37.80, 44.65)	−0.516	0.606
PA (mg/L)	200.55 (162.80,256.00)	205.05 (166.55,257.90)	−0.262	0.794
HGB (g/L)	133.00 (119.50,145.00)	122.00 (111.00,136.00)	4.696	<0.001
WB (10^9/L)	9.20 (7.03, 11.42)	9.47 (7.32, 11.64)	−0.248	0.804
NEU (10^9/L)	7.29 (5.24, 9.90)	7.11 (5.48, 9.81)	−0.206	0.837
CRP (10^9/L)	6.42 (3.72, 13.10)	6.56 (3.57, 9.20)	−1.019	0.308
LYM (10^9/L)	1.32 (1.10, 1.82)	1.42 (1.10, 1.89)	−0.781	0.435

Data are presented as *n* (%), median (Q1, Q3), or mean ± SD. Statistical tests: Pearson's chi-square test; Mann–Whitney U test. TP, total protein; ALB, albumin; PA, prealbumin; HGB, hemoglobin; WBC, white blood cell; NEU, neutrophil; CRP, C-reactive protein; LYM, lymphocyte.

^1^*n* (%), Median (Q1, Q3), or mean ± SD; ^2^Pearson's Chi-squared test; Fisher's exact test; Wilcoxon rank sum test.

### 3.2 Univariate analysis of frailty in patients with PAD

Significant differences were observed between the frailty and non-frailty groups in terms of age, type of medical insurance, disease stage, number of comorbidities, hemoglobin levels, ADL, and social support, with all differences being statistically significant (*P* < 0.05) (Table 1).

### 3.3 Logistic regression analysis of frailty in patients with PAD

Frailty status was used as the dependent variable (No = 0; Yes = 1), and seven variables identified as statistically significant (*P* < 0.05) in the univariate analysis—including age, type of medical insurance, disease stage, number of comorbidities, hemoglobin level, ADL, and social support—were included as independent variables in a binary logistic regression analysis. Continuous variables were entered using raw values, while the coding scheme for categorical variables is provided in Table 2. Multicollinearity diagnostics indicated no multicollinearity among the variables. The logistic regression results identified age, hemoglobin level, number of comorbidities, and ADL as independent predictors of frailty in patients with PAD (*P* < 0.05) (Table 3).

**Table 2:** Assignment methods for independent variables.

Variables	Value assignment approach
Age	50∼70 = 1 71∼80 = 2 >80 = 3
Medical insurance statue	urban employees = 1 urban residents = 2
Fontaine stage	Ⅱa stage = 1 Ⅱb stage = 2 Ⅲ stage = 3 Ⅳ stage = 4
Number of comorbidities	0 = 1 1∼3 = 2 >3 = 3
ADL	Self-care = 1 Mild = 2 Moderate = 3 Severe = 4
Social support	Low level = 1 Moderate level = 2 High level = 3
HGB	Plugging in the original values

**Table 3:** Variables results of univariate logistic regression analysis for frailty occurrence in patients with PAD (*n* = 350).

Variables	*β*	S.E	Wald*χ*^2^	*P*-value	OR (95%CI)
Constant	−11.09	1.907	33.728	–	–
Age					
50∼70		–	–	–	–
71∼80	0.683	0.376	1.821	0.069	1.982 (0.963∼4.201)
>80	1.502	0.476	3.165	0.002	4.521 (1.802∼11.73)
Number of comorbidities					
0		–	–	–	–
1∼3	1.523	0.687	2.223	0.027	4.593 (1.347∼21.542)
>3	1.657	0.744	2.321	0.026	5.221 (1.349∼26.754)
HGB	−0.055	0.009	6.274	<0.001	0.910 (0.904∼0.912)
ADL					
Full independence		–	–	–	–
Mild dysfunction	0.294	0.620	0.520	0.601	1.380 (0.413∼4.776)
Moderate dysfunction	0.486	0.671	2.523	0.012	5.435 (1.523∼21.624)
Severe dysfunction	2.097	0.623	2.839	0.005	5.842 (1.796∼20.903)

β, regression coefficient; S.E, standard error; OR, odds ratio; CI, confidence interval.

### 3.4 Predictive model development and validation

**Figure 2:**
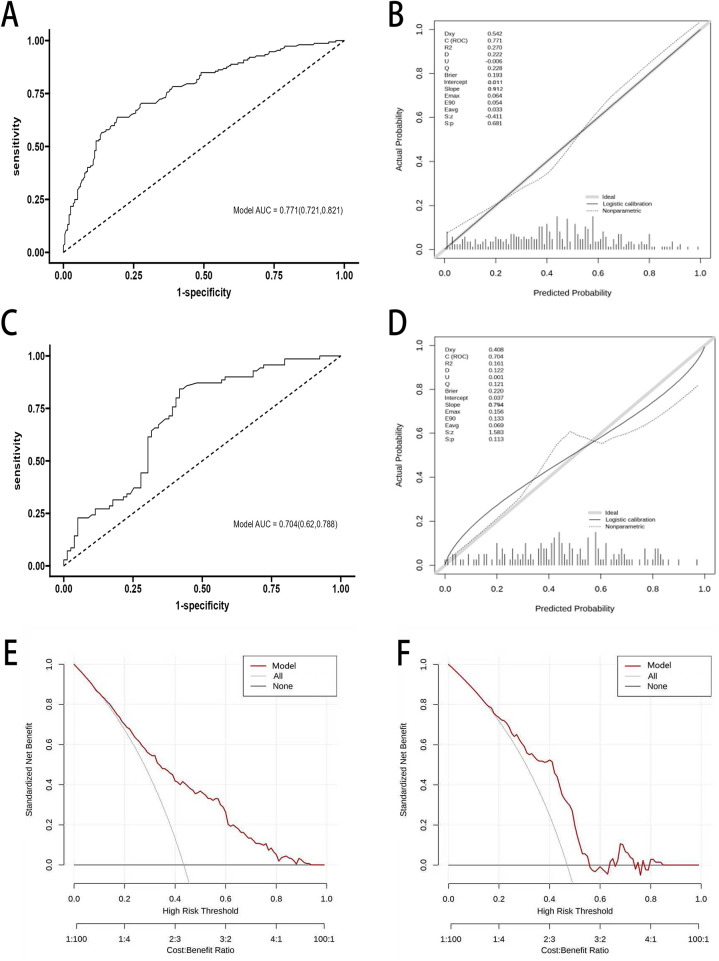
Performance of the frailty prediction model for PAD. **(A)** ROC curve of the frailty prediction model in the training set. **(B)** Calibration curve of the frailty prediction model in the training set. **(C)** ROC curve of the frailty prediction model in the validation set. **(D)** Calibration curve of the frailty prediction model in the validation set. **(E)** DCA of the frailty prediction model in the training set. **(F)** DCA of the frailty prediction model in the validation set.

Based on the predictors identified by logistic regression, their corresponding *β* coefficients, and the constant term, a frailty risk prediction model for older patients with PAD was established as follows: Logit(*P*) = −11.09 + 0.683 × age (71–80 years) + 1.502 × age (>80 years) + 1.523 × number of comorbidities (1–3) + 1.657 × comorbidities (>3)−0.055 × hemoglobin + 0.294 × ADL (mild dysfunction) + 0.486 × ADL (moderate dysfunction) + 2.097 × ADL (severe dysfunction).In the training set, the model showed an AUC of 0.771 (95% CI: 0.721–0.821), with a sensitivity of 0.788 and a specificity of 0.808, the positive predictive value is 0.719, and the negative predictive value is 0.804(Figure 2A). The Hosmer–Lemeshow goodness-of-fit test yielded *χ*^2^ = 7.967 and *P* = 0.435 (*P* > 0.05). The Brier score was 0.193, with a slope of 0.912 and an intercept of 0.011, indicating good calibration (Figure 2B). In the validation set, the model yielded an AUC of 0.704 (95% CI: 0.626–0.788), with a sensitivity of 0.743 and a specificity of 0.682, the positive predictive value is 0.614, and the negative predictive value is 0.807(Figure 2C). The Hosmer–Lemeshow test showed *χ*^2^ = 9.642 and *P* = 0.291 (*P* > 0.05). The Brier score was 0.220, with a slope of 0.794 and an intercept of 0.037, suggesting acceptable calibration (Figure 2D). Decision curve analysis (DCA) was used to evaluate the clinical utility of the model. The results showed relatively wide threshold probability ranges in both sets. In the training set, DCA indicated a higher net clinical benefit when the threshold probability ranged from 10% to 80%. In the validation set, a higher net clinical benefit was observed when the threshold probability ranged from 20% to 74% (Figures 2E,F).

### 3.5 Nomogram for predicting the risk of frailty in patients with PAD

**Figure 3:**
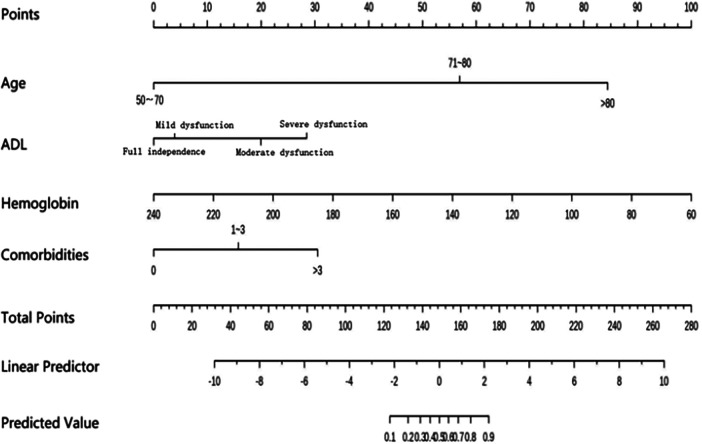
Nomogram risk model of frailty in patients with PAD.

Based on the results of the multivariable logistic regression analysis, a quantitative frailty risk prediction nomogram was developed in RStudio, incorporating four variables: age, hemoglobin, number of comorbidities, and ADL (Figure 3). The nomogram was used to estimate the probability of frailty. Briefly, points are assigned to each predictor according to its relative contribution to the outcome in the model; the points for all predictors are then summed to obtain a total score. Through a function-based transformation, the relationship between the total score and the predicted probability of the outcome (frailty in this study) is displayed in an intuitive manner, allowing calculation of the individual risk.

To use the nomogram, “Points” represents the score for each predictor, and “Total points” is the sum of the scores across all predictors. A vertical line drawn downward from the “Total points” axis yields the corresponding predicted probability of the outcome. For example, for a patient with PAD aged 80 years (about 56 points), with moderate ADL dysfunction (about 20 points), hemoglobin of 120 g/L (about66 points), and more than three comorbidities (about 30 points), the total score is approximately 172 points, corresponding to an estimated 90% probability of frailty. Higher total scores indicate a higher risk of frailty.

## 4 Discussion

### 4.1 Prevalence of frailty in patients with PAD

This study identified age, hemoglobin, number of comorbidities, and ADL as independent risk factors for frailty in patients with PAD. The prevalence of frailty among patients with PAD in this study was 43.4%, which is higher than the 26.1% reported by Nishikawa et al., but lower than the 52% reported by Houghton et al. (8, 22). These discrepancies may be attributed to differences in study populations and assessment tools. Participants in both of the aforementioned studies had end-stage chronic limb-threatening ischemia, whereas this study included hospitalized patients with PAD at Fontaine stages II to IV. Furthermore, the study used the Tilburg Frailty Indicator (TFI), while the other two studies used the Clinical Frailty Scale (CFS) and the Frailty Phenotype Model, respectively. The prevalence of frailty is highly dependent on the assessment tools used, and variation in tools contributes to inconsistent prevalence rates across studies. This highlights the need for vascular healthcare providers to emphasize frailty assessment in patients with PAD and to carefully consider the accuracy and feasibility of the chosen frailty assessment instruments. The use of standardized tools is crucial for effectively screening frailty risk in patients with PAD. Frailty is not merely a conceptual construct; it is a decisive factor that can independently and substantially influence vascular surgical decision-making, wound healing, and amputation and survival rates. Frail patients have reduced physiological reserve, are more susceptible to postoperative complications, and experience a slower recovery process. Evidence indicates that timely implementation of preoperative frailty screening and optimization pathways can effectively reduce 30-day readmission rates after vascular surgery (23). Frailty is a potentially reversible condition. Early identification and targeted intervention can improve risk stratification, inform clinical decision-making, guide personalized treatment strategies, minimize complications and adverse outcomes, promote functional recovery, and improve patient prognosis.

### 4.2 Risk factors associated with frailty in patients with PAD

This study found a significant positive correlation between age and frailty in patients with PAD, identifying age as a major risk factor—consistent with previous findings (13). Increasing age is associated with increased levels of inflammatory biomarkers and progressive dysregulation of immune mechanisms. Research suggests that immune dysfunction and chronic inflammation are key pathogenic drivers in the development of frailty (24). Chronic inflammation adversely affects skeletal muscle mass and strength in the elderly by inhibiting protein synthesis and enhancing protein degradation. Moreover, inflammatory biomarkers are closely linked to frailty and can influence hormone secretion and metabolism, ultimately leading to increased resting energy expenditure (25). Additionally, older patients with PAD tend to have reduced physiological reserve and impaired function across multiple organ systems, leading to diminished stress resilience and a higher susceptibility to frailty. Overall, age-related immune dysregulation, declining physiological resistance, and hormonal alterations collectively increase the risk of frailty. These findings underscore the importance for vascular healthcare professionals to strengthen early screening for frailty among older patients with PAD. Timely interventions and targeted nursing strategies can significantly improve their quality of life, delay the progression of frailty, and reduce the risk of complications.

The predictive model developed in this study identified hemoglobin as an independent risk factor for frailty in patients with PAD. Lower hemoglobin levels were associated with a higher risk of frailty, which is consistent with findings from previous studies (26, 27). Hemoglobin, a key protein component of red blood cells, serves as a biomarker for both anemia and nutritional status. Its primary role is to facilitate efficient oxygen transport, making it essential for aerobic metabolism and the maintenance of internal homeostasis. Reduced hemoglobin levels lead to decreased tissue oxygenation, which can impair muscle and organ function, contribute to fatigue, and ultimately increase vulnerability to frailty (28, 29). A cross-sectional study conducted among older adults in Asian communities demonstrated that for every 1 g/L increase in hemoglobin, the odds of frailty decreased by 6% after adjusting for covariates (OR=0.94, 95% CI: 0.90–0.99) (30). Anemia is highly prevalent among patients with PAD. One study found that approximately 52.3% of patients with advanced chronic limb-threatening ischemia exhibited anemia (31). These findings suggest that hemoglobin concentration may serve as a valuable indicator for identifying frailty in patients with PAD. Regular monitoring of hemoglobin levels, combined with personalized nutritional support through a multidisciplinary approach, may help to correct anemia, ensure adequate nutrient intake, improve overall health status, and prevent the progression of frailty. However, hemoglobin was measured only once at enrollment in the present study. Therefore, we were unable to distinguish the long-term effects of chronic anemia from the short-term impact of an acute hemoglobin decline (e.g., due to a bleeding event). Nevertheless, given that anemia in patients with PAD is often chronic, we speculate that baseline hemoglobin primarily reflects an underlying chronic pathological state that may share biological pathways with frailty (32). Future studies should incorporate longitudinal, repeated hemoglobin measurements to better clarify how dynamic changes in hemoglobin relate to the onset and progression of frailty.

The predictive model developed in this study identified the coexistence of multiple chronic diseases as a significant risk factor for frailty in patients with PAD. This finding aligns with previous research results (33). PAD is an age-related disease, and most older adults have concomitant chronic conditions. Patients with PAD frequently present with multiple chronic diseases. In this study, comorbidities including diabetes mellitus, hypertension, coronary artery disease, stroke, and chronic kidney disease were considered. Although each condition has its distinct mechanisms, their fundamental driver may reflect a shared underlying process, namely “inflammaging” (34). Inflammaging refers to a chronic, low-grade inflammatory state associated with the aging process (35). A meta-analysis published in 2019 showed that older adults with two or more chronic diseases had a 3–5-fold higher risk of frailty than those without a comorbidity burden, and when the number of chronic diseases reached three or more, the majority of individuals developed frailty to varying degrees (36). Based on this evidence, we categorized comorbidity burden into three groups: no comorbidity burden, multimorbidity burden, and high multimorbidity burden. The higher the comorbidity burden, the greater the risk of frailty in patients with PAD. This may be because the coexistence of multiple chronic diseases places multiple organ systems in a state of long-term exhaustion, disrupts physiological homeostasis, weakens the ability to withstand external stressors, and increases frailty risk. Frailty represents the cumulative loss of subclinical reserves across multiple systems and may be accompanied by multimorbidity and chronic diseases, leading to persistent functional decline (37). This supports an established view that, with advancing age, the gradual development of frailty is not merely a consequence of chronological age itself, but more likely results from the combined effects of biological aging processes and the accumulation of multimorbidity. Artaç et al. used the Naples Prognostic Score to predict long-term outcomes after percutaneous intervention in patients with PAD, clearly highlighting the synergistic roles of inflammation (aging and comorbidity burden) and nutritional status in driving PAD progression; this score was also strongly associated with all-cause mortality and amputation after endovascular treatment (38). Our findings are highly consistent with this evidence: in our model, nutritional status and comorbidity burden jointly contribute to the occurrence and progression of frailty, thereby indirectly influencing prognosis in patients with PAD.

The Barthel Index is an important measure of ADL. Our prediction model demonstrated a significant association between ADL and frailty in patients with PAD. Previous studies have demonstrated a strong correlation between frailty and functional decline, establishing ADL as a key predictive factor for frailty (39). Impaired performance in ADL not only reflects diminished self-care ability but may also negatively impact dietary habits, thereby increasing the risk of malnutrition. Moreover, functional impairment is often accompanied by reduced physical activity, which leads to muscle weakness and decreased bone density—factors that elevate the risk of sarcopenia and osteoporosis, ultimately increasing the likelihood of frailty (40). Additionally, patients with impaired performance in ADL may experience limited mobility and reduced social engagement, contributing to social isolation and diminished social support, both of which are known to exacerbate frailty risk (41). Therefore, vascular surgeons and nursing staff should systematically assess the ADL status of patients with PAD to facilitate early identification of frailty. Multidisciplinary interventions should be implemented to help patients maintain or improve their self-care abilities. Patients should be encouraged to engage in daily physical activities within their capabilities, explore new experiences, and actively participate in social interactions. Such strategies may delay physical decline, prevent further frailty progression, and enhance overall health outcomes.

In our univariate analyses, disease stage, type of medical insurance, and social support were all associated with frailty; however, they were not retained as independent predictors in the multivariable model. This may be explained as follows. Although disease stage was included in the analyses, patients with stage III–IV disease accounted for more than 80% of the total sample. This substantial imbalance in disease-stage distribution may have reduced the ability of disease stage to demonstrate an independent predictive effect in the regression model. Therefore, this model may not be applicable to patients with mild or asymptomatic PAD. Social support and health insurance status are key social determinants of health outcomes. In this study, the effects of insurance type and social support may have been “mediated” by other physiological and comorbidity-related variables already included in the model. Insurance type is often closely related to access to healthcare resources, continuity of treatment, and socioeconomic status, and may therefore influence overall health status and frailty risk indirectly. Patients with poor social support may also find it more difficult to maintain a healthy diet and engage in regular physical activity, behaviors that can ultimately contribute to malnutrition and sarcopenia (42). These downstream consequences may have been captured by objective indicators in our model (e.g., hemoglobin level) and comorbidity burden, thereby attenuating the independent contribution of social support in the multivariable analysis.

However, several limitations should be noted. First, the data were obtained from a single-center study conducted at one hospital in Southwest China, which may limit the model's generalizability. External validation using populations from other regions is warranted to evaluate its applicability across different cultural and socioeconomic contexts. Second, the study did not perform subgroup analyses or assess publication bias; variations in disease stage and PAD severity may influence the findings. Third, this was a cross-sectional study. Previous studies have identified several factors influencing frailty, including advanced age, malnutrition, inflammation, physical inactivity, comorbid chronic diseases, depressive symptoms, and limitations in activities of daily living (ADL) (15–17). In our study, priority was given to variables that were available in our dataset. However, certain factors reported in the literature, such as specific biomarkers and psychological assessments requiring evaluation by mental health professionals, were not included due to data unavailability and the need for specialized assessments. We acknowledge the limitations of our variable selection and hope that future research, through prospective data collection and multidisciplinary collaboration, can incorporate additional biological and psychosocial factors to further elucidate the determinants of frailty. Fourth, some variables (e.g., high-sensitivity C-reactive protein) had a relatively high proportion of missing data (7.8%). Although multiple imputation was applied, the imputed values rely on the assumption that data are missing at random; if the missingness was not at random, bias may have been introduced. Finally, most participants in this study had symptomatic PAD, and patients with mild or asymptomatic PAD in outpatient settings were not included. Future research should enroll patients across the full spectrum of PAD stages and use stratified analyses to characterize frailty by disease stage and to inform more individualized and precise intervention strategies.

## 5 Conclusion

In this study, age, hemoglobin, number of comorbidities, and ADL were incorporated into a nomogram model to predict the risk factors for frailty in patients with PAD. Evaluation of the model's discrimination and calibration demonstrated good predictive performance in terms of both discriminatory ability and calibration accuracy. Therefore, the nomogram developed in this study provides a theoretical foundation for understanding frailty in Patients with PAD and offers scientific support for vascular healthcare professionals in effectively identifying frailty and formulating prevention and intervention strategies.

## Data Availability

The original contributions presented in the study are included in the article/Supplementary Material, further inquiries can be directed to the corresponding author.
